# Supplementary material for: Risk factors for sacrococcygeal pilonidal sinus: a systematic review and meta-analysis supplemented by genetic causal assessment
Source: Front Surg. 2026 Jan 7;12:1718589. doi: 10.3389/fsurg.2025.1718589 (PMC12819706; doi:10.3389/fsurg.2025.1718589)
Supplement: Supplementary file 2 [file Datasheet2.zip › Supplementary Data 2/MR_pipeline_p5e-8/leaveone_plots/ukb-d-1747_2.finngen_R12_L12_PILONIDALCYST.pdf]

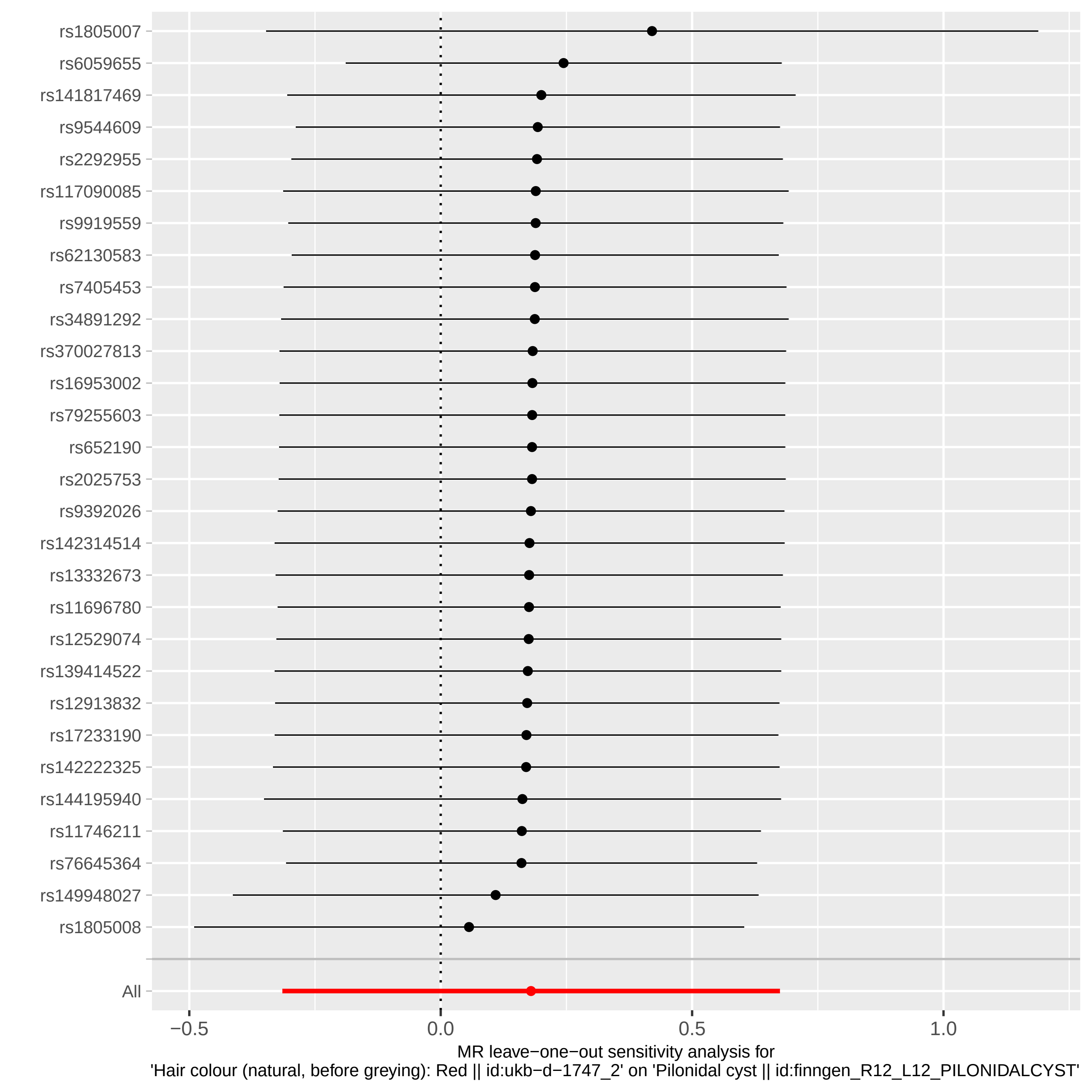

rs1805007
rs6059655
rs141817469
rs9544609
rs2292955
rs117090085
rs9919559
rs62130583
rs7405453
rs34891292
rs370027813
rs16953002
rs79255603
rs652190
rs2025753
rs9392026
rs142314514
rs13332673
rs11696780
rs12529074
rs139414522
rs12913832
rs17233190
rs142222325
rs144195940
rs11746211
rs76645364
rs149948027
rs1805008
All
−0.5
0.0
0.5
1.0
MR leave−one−out sensitivity analysis for
'Hair colour (natural, before greying): Red || id:ukb−d−1747_2' on 'Pilonidal cyst || id:finngen_R12_L12_PILONIDALCYST'
